# Effect of dexmedetomidine on lung ischemia-reperfusion injury

**DOI:** 10.3892/mmr.2013.1867

**Published:** 2013-12-17

**Authors:** LILI JIANG, LI LI, JINMEI SHEN, ZEYOU QI, LIANG GUO

**Affiliations:** Department of Anesthesiology, Second Xiang-Ya Hospital, Xiang-Ya Medical College, Central South University, Changsha, Hunan 410011, P.R. China

**Keywords:** dexmedetomidine, ischemia-reperfusion injury, interleukin-6, tumor necrosis factor-α

## Abstract

Dexmedetomidine, a specific selective α_2_-adrenergic agonist, does not only have the characteristics of being a sedative and analgesic, but also exhibits a protective role in brain ischemia-reperfusion injury and inhibits the inflammation in animals with sepsis. The objective of the present study was to investigate whether dexmedetomidine is capable of attenuating rat pulmonary damage induced by ischemia-reperfusion injury, which is a type of acute sterile lung injury. Sprague-Dawley rats were randomly assigned into six groups: The sham-operated (sham) group, the lung ischemia-reperfusion (I/R) group, intravenous injection of dexmedetomidine 2.5 μg/kg/h (Dex2.5) or 5 μg/kg/h (Dex5) for 1 h prior to ischemia, combination of α_2_-adrenergic antagonist yohimbine prior to dexmedetomidine pre-treatment (Dex+Yoh) and pre-administration of yohimbine alone (Yoh) prior to ischemia. Lung injury was assessed by the histopathological changes, arterial blood gas, wet/dry (w/d) weight ratio and myeloperoxidase (MPO) activity of the lung. The concentration of tumor necrosis factor-α (TNF-α), interleukin-6 (IL-6) and monocyte chemoattractant protein-1 (MCP-1) in bronchoalveolar lavage fluid (BALF) was measured by an enzyme-linked immunosorbent assay. The expression of toll-like receptor-4 (TLR4) and myeloid differentiation factor 88 (MyD88) mRNA in the lung were determined by quantitative PCR, and phosphorylated levels of c-Jun N-terminal kinase (JNK) and extracellular signal-regulated kinase (ERK)1/2 were determined by western blotting. Pre-treatment with dexmedetomidine significantly reduced the lung injury, w/d weight ratio and MPO activity, and decreased the concentration of TNF-α, IL-6 and MCP-1 in BALF compared with the I/R group. The expression of TLR4 and MyD88 mRNA and the levels of phosphorylated JNK and ERK1/2 in the lung tissue were markedly downregulated by intravenous injection of dexmedetomidne for 1 h prior to lung I/R. The protective effects of dexmedetomidine on the lung were not completely reversed by the α_2_-adrenergic antagonist, yohimbine. Pre-treatment with dexmedetomidine is capable of reducing pulmonary damage and inhibiting sterile inflammation induced by lung I/R injury. TLR4/MyD88/mitogen-activated protein kinase (MAPK) signaling is involved in the protective mechanism of dexmedetomidine through α_2_-adrenoceptor independence.

## Introduction

Lung ischemia-reperfusion injury (LIRI), a form of acute sterile lung injury, remains a frequent complication resulting in morbidity and mortality in lung transplantation (1), cardiopulmonary bypass (2), trauma (3), pulmonary embolism (4) and resuscitation from hemorrhagic shock (5). Early activation of the alveolar macrophages, the release of various proinflammatory molecules and a large amount of neutrophil accumulation are critical in the pathophysiology of LIRI, which leads to excessive and uncontrolled inflammation and pulmonary tissue damage. Thus, it is beneficial to inhibit the upregulation of pulmonary inflammation for suppression of the development of lung injury (6).

Dexmedetomidine is a selective α_2_-adrenergic agonist with sedative, antianxiety, analgesic-sparing, sympatholytic and hemodynamic stability characteristics (7). In addition, it has been demonstrated to reduce endotoxine-induced systemic inflammatory responses, inhibit upregulation of inflammatory cytokines, including tumor necrosis factor-α (TNF-α), interleukin-1β (IL-1β), IL-6 and macrophage inflammatory protein-2 (MIP-2); and relieve acute organ injuries in rats and patients with sepsis (8,9). Moreover, dexmedetomidine has been reported to significantly attenuate the pulmonary inflammation in ventilator-induced lung injury in a rat model (10). Although the anti-inflammatory capacity of dexmedetomidine has been demonstrated, the effect of dexmedetomidine on the inflammatory molecules, including TNF-α, IL-6 and monocyte chemoattractant protein (MCP)-1, correlated with LIRI remains unclear. Therefore, it was hypothesized that dexemedetomidine may attenuate the production of proinflammatory chemokines and cytokines and protect the lung from acute I/R injury.

Toll-like receptors (TLRs) are a family of transmembranal proteins that are critical in the regulation of the inflammatory and innate immune responses (11). Among these receptors, TLR4, widely expressed on immune cells and non-immune cells, including dendritic cells, neutrophils, macrophages, endothelial cells, epithelial cells and natural killer cells, specifically recognizes endogenous molecules released from damaged or ischemic tissues termed danger-associated molecular patterns (DAMPs), and then activates multiple intracellular signaling systems, including the mitogen-activated protein kinase (MAPK) family [extracellular signal-regulated kinase (ERK), c-Jun N-terminal kinase (JNK) or p38], and the nuclear factor κB (NF-κB) pathways, all of which are key regulators of the inflammatory responses during I/R of the organs (3). In the mouse LIRI model, the upregulation of TLR4 mRNA expression in alveolar macrophages is vital in the generation of the early inflammatory response to LIRI, which is paralleled by the observation of lung neutrophil recruitment in histological findings and the generation of cytokines and chemokines (12). Shi *et al* (13) identified that dexmedetomidine provides protection against LPS-induced acute lung injury through the TLR4/NFκB signaling pathway, and reduces the level of proinflammatory factors in lung homogenates, as well as the lung damage observed in histological findings. Therefore, the present study was conducted to determine whether the pre-administration of dexemedetomidine may provide a significant effect on relieving pulmonary damage induced by I/R, and to observe whether TLR4 relevant signaling pathways may be involved in the effects of dexemedetomidine on LIRI.

## Materials and methods

### Animals

The following investigations were performed using a protocol approved by the Animal Care and Use Committee of the Xiang-Ya Medical College of the Central South University (Changsha, China). Adult male Sprague-Dawley rats (220–270 g) were housed in individual cages in a temperature- and humidity-controlled room with a 12-h light/dark cycle, and acclimated for one week prior to the study. All rats were fed standard pellet food and water *ad libitum*.

### Induction of lung I/R, as described previously (3)

All the rats were anesthetized with pentobarbital sodium (50 mg/kg, intraperitoneal) and atropine (0.25 mg, intramuscular). A tracheotomy was performed following anesthesia, and the trachea was intubated with a 14-gauge intravenous (i.v.) catheter (B. Braun Melsungen AG, Melsungen, Germany). The rats were then mechanically ventilated with a small animal ventilator (Beijing Zhongxi Yuanda Technology Inc., Beijing, China), adjusting the tidal volume to 10 ml/kg of room air, the respiratory rate to 50 breaths/min and the inspiratory/expiratory ratio to 1:1. The body temperature was maintained at 38–40°C using a heating pad. An intravenous 24-gauge catheter was then installed in the tail vein for drug and saline administration. Subsequently, a left anterolateral thoracotomy was undertaken through the fourth intercostal space, with a right lateral position. At 5 min after the injection of heparin (100 U/kg, i.v.), the left pulmonary hilum, including the left main bronchus, artery and vein, was occluded at the end of an expiration/breath with a non-invasive microvascular clip for 1 h. At the end of the ischemic period, the clip was removed and the lung regained ventilation and reperfusion for 2 h. The rats of the sham group underwent sham surgery consisting of a thoracotomy without clamping of the left pulmonary hilum. Maintenance fluids (0.9% saline) were administered at 1.0 ml/h by tail vein for the duration of the I/R phase. At the end of each experiment, all the rats were sacrificed by bleeding in the right ventricle of the heart.

### Experimental protocol and drug administration

A total of 72 rats were randomly allocated to six groups (n=12 per group) as follows: i) The sham group, saline-treatment (1 ml/h i.v.) without I/R; ii) the I/R group, saline-treatment (1 ml/h i.v.) for 1 h prior to I/R; iii) the Dex2.5 group, i.v. infusion of dexmedetomidine at a dose of 2.5 μg/kg/h for 1 h prior to I/R; i.v.) the Dex5 group, i.v. infusion of dexmedetomidine at a dose of 5 μg/kg/h for 1 h prior to I/R; i.v.) the Dex+Yoh group, i.v. infusion of yohimbine (1.0 mg/kg, injection process lasting no less than 5 min) followed by infusion of dexmedetomidine at a dose of 5 μg/kg/h for 1 h prior to I/R and v) the Yoh group, i.v. infusion of yohimbine (1.0 mg/kg, injection process lasting no less than 5 min) followed by infusion of saline at 1 ml/h for 1 h prior to I/R.

### Arterial blood gas (ABG) analysis

At the end of each experiment, 0.5 ml blood was drawn from the left ventricle of the heart. ABG levels were immediately measured with a blood gas analyzer (GEM Premier 3000; Instrumentation Laboratory Co., Bedford, MA, USA).

### Bronchoalveolar lavage (BAL)

For six rats of each group, the right main bronchus was tied and the left lung was lavaged five times with 10 ml cold sterile saline (14). Once the BAL fluid (BALF) had been collected and centrifuged at 3,200 × g, 4°C (Eppendorf 5840R; Eppendorf, Hamburg, Germany), the BALF supernatant was obtained for further analysis of the levels of TNF-α, IL-6 and MCP-1 by enzyme-linked immunosorbent assay (ELISA; TNF-α, IL-6 and MCP-1 ELISA kits; R&D Systems, Inc., Minneapolis, MN, USA).

### Perfusion fixation and histopathological analysis

The left lung tissues from the six rats of each group were perfused with 4% formaldehyde and then removed. The formaldehyde-infused left lungs were embedded in paraffin wax, sectioned (5 μm) and stained with hematoxylin and eosin. The histological changes were scored by a pathologist in a blinded manner. The lung injury was classed using a score of 0 to 12 (grade 0, 1, 2 or 3 standing for normal, mild, moderate or severe, respectively) for intra-alveolar edema, intra-alveolar hemorrhage and neutrophil infiltration (15).

### Wet/dry (w/d) weight ratio and myeloperoxidase (MPO) activity assay

For the other six rats of each group, the left main bronchus was tied and the left lung was removed after the rats had been sacrificed. The upper and lower lobes of the left lung were divided, and the lower lobes were snap-frozen in liquid nitrogen and stored at −70°C for the MPO activity assay and for total RNA and protein extraction. The left upper lobe of the lung tissue was immediately weighed following harvest to obtain the wet weight and again following desiccation in an oven at 60°C for 48 h to obtain the dry weight. The lung water content was assessed by the w/d ratio. In addition, a 100 mg snap-frozen left lower lobe lung sample was homogenized in cold saline, and the MPO activity was measured with an MPO assay kit, according to the manufacturer’s instructions (Nanjing Jiancheng Bioengineering Institute, Nanjing, Jiangsu, China). The results are expressed as units per gram of protein.

### Quatitative PCR (qPCR) assay of TLR4 and MyD88 mRNA

The mRNA expression of TLR4 and MyD88 was analyzed by qPCR. The total RNA was extracted by homogenization of the lung tissue in TRIzol reagent (Invitrogen Life Technologies, Carlsbad, CA, USA). In total, 5 μg total RNA was reverse-transcribed into cDNA, and the PCR reaction mixtures were prepared using SYBR Green qPCR master mix (Toyobo Co., Ltd., Osaka, Japan). β-actin, a housekeeping gene, was used as the internal control. Primers (Huada Gene, Beijing, China) were designed with sequences as follows: Forward: 5′-GAATGAGGACTGGGTGAGAAAC-3′ and reverse: 5′-ACCAACGGCTCTGGATAAAGT-3′ for TLR4; Forward: 5′-ACCGCATCGAGGAGGACTG-3′ and reverse: 5′-CTGTGGGACACTGCTCTCCA-3′ for MyD88; and forward: 5′-AGGCCCCTCTGAACCCTAAG-3′ and reverse: 5′-CCAGAGGCATACAGGGACAAC-3′ for β-actin. The relative expression levels of TLR4 and MyD88 mRNA in the lung tissues were determined by the 2^−ΔΔCT^ method (16) following normalization to the housekeeping gene, β-actin.

### Western blotting

Snap-frozen lung tissue (50 mg) was homogenized and centrifuged at 12,000 × g at 4°C for 20 min, and the supernatant was collected. The protein concentration was determined by a bicinchoninic acid protein assay (Proteintech, Wuhan, Hubei, China). Samples with equal quantities of protein were mixed with 5X SDS sample buffer (Dingguo Changsheng Biotechnology Co., Ltd, Beijing, China) and then boiled for 5 min. Aliquots of the samples were separated on a 10% Tris-glycine gel and electrophoresed. Following transfer to nitrocellulose membranes, the samples were blocked by 5% skimmed milk powder in Tris-buffered saline with Tween 20 (30 mM Tris-HCl, 125 mM NaCl and 0.1% Tween-20) for 1 h at room temperature, and then incubated overnight at 4°C with rabbit polyclonal antibodies against phosphorylated ERK (P-ERK) or total ERK and JNK (Santa Cruz Biotechnology, Inc., Santa Cruz, CA, USA). Subsequent to being washed, the primary antibodies were counterstained with horseradish peroxidase-conjugated goat-anti-rabbit IgG antibody (Cell Signaling Technology, Inc., Danvers, MA, USA), visualized with enhanced chemiluminescence detection reagents (Merck Milipore, Darmstadt, Germany) and finally exposed to photographic film for a suitable length of time. The images were analyzed using Image J software, and the ratios of P-ERK/ERK and P-JNK/JNK provided a measurement of the ERK and JNK phosphorylation levels.

### Statistical analysis

All data are presented as the mean ± standard deviation. The experimental results were analyzed by SPSS 17.0 software (SPSS, Inc., Chicago, IL, USA). The differences among groups were assessed by one-way analysis of variance with the Bonferroni test for variances. P<0.05 was considered to indicate a statistically significant difference.

## Results

### Lung histology

**Figure 1:**
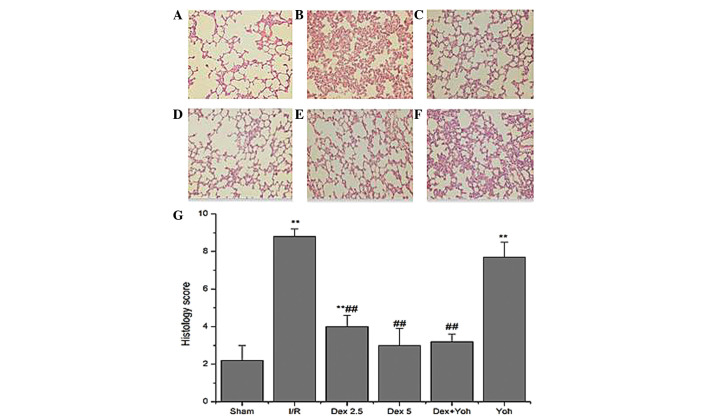
Microscopic findings from the rat left lung tissues stained with hematoxylin and eosin (magnification, ×200). The (A) sham (B) I/R (C) Dex2.5 (D) Dex5 (E) Dex+Yoh and (F) Yoh groups. (G) The severity of lung injury was scored and the data are presented as the mean ± standard deviation. ^**^P<0.01 vs. the sham group and ^##^P<0.01 vs. the I/R group. Dex, dexmedetomidine; Dex 2.5, 2.5 μg/kg/h Dex; Dex 5, 5 μg/kg/h; Yoh, yohimbine; DexI/R, ischemia-reperfusion.

The histological analysis showed minimal lung injury in the rats of the sham group (Fig. 1A) and severe lung injury in the rats of the I/R (Fig. 1B) and Yoh (Fig. 1F) groups. By contrast, the lung tissues harvested from the rats of the Dex2.5 (Fig. 1C), Dex5 (Fig. 1D) and Dex+Yoh (Fig. 1E) groups revealed mild to moderate damage compared with that of the sham group, respectively. The lung injury scores paralleled the histological findings. The lung injury scores of the I/R (8.8±0.4; P<0.01) and Yoh (7.7±0.8; P<0.01) groups were significantly higher compared with that of the sham group (2.2±0.8), whereas those of the Dex2.5 (4.0±0.6; P<0.01), Dex5 (3.0±0.9; P<0.01) and Dex+Yoh (3.2±0.4; P<0.01) groups were significantly lower compared with that of the I/R group, but there was no significant difference among them (Fig. 1G).

### ABG data, w/d weight ratio and MPO activity

**Figure 2:**
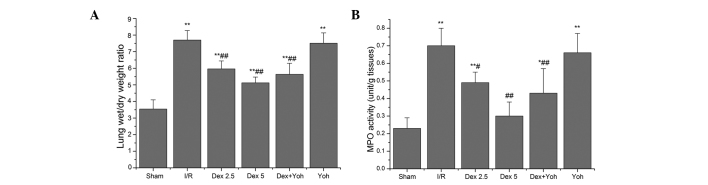
Ratio of (A) wet/dry weight and (B) MPO activity assays in lung tissues harvested from rats of the six groups. The data are presented as the mean ± standard deviation. ^*^P<0.05 and ^**^P<0.01 vs. the sham group; ^#^P<0.05 and ^##^P<0.01 vs. the I/R group. MPO, myeloperoxidase; Dex, dexmedetomidine; Dex 2.5, 2.5 μg/kg/h Dex; Dex 5, 5 μg/kg/h; Yoh, yohimbine; I/R, ischemia-reperfusion.

The pH, PaO_2_, PaCO_2_ and base excess values exhibited no significant differences among the six groups (Table I). The lung w/d weight ratio was measured following reperfusion to evaluate the I/R-induced lung edema. In the I/R group, the ratio (7.70±0.57) was significantly increased compared with that of the sham group (3.54±0.56; P<0.01). The ratio was significantly decreased following pre-administration of dexmedetomidine (Dex2.5 group, 5.96±0.48; and Dex5 group, 5.12±0.35) prior to lung ischemia compared with that of the I/R group (all P<0.01). However, yohimbine failed to reverse the effect of dexmedetomidine on the lung w/d weight ratio, and there was no significant difference in the w/d ratio between the Yoh (7.51±0.62; P>0.05) and I/R groups (Fig. 2A).

MPO activity, a biochemical marker of neutrophil infiltration, rose to 0.70±0.10 in the lung of the I/R group compared with that of the sham group (0.23±0.06; P<0.01). Pre-treatment with dexmedetomidine resulted in a significant reduction in the lung MPO activity of the Dex2.5 (0.49±0.06; P<0.05) and Dex5 (0.30±0.08; P<0.01) groups compared with that of the I/R group. However, yohimbine failed to reverse the effect of dexmedetomidine on the lung MPO activity, and there was no significant difference in MPO activity between the Yoh (0.66±0.11; P>0.05) and I/R groups (Fig. 2B).

### Effects of dexmedetomidine on the concentration of TNF-α, IL-6 and MCP-1 in BALF

**Figure 3:**
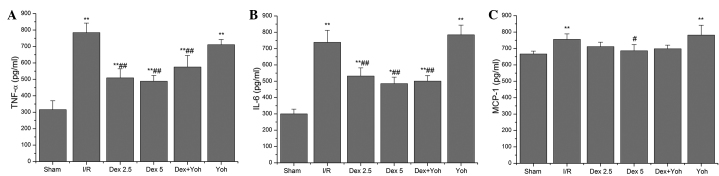
ELISA analysis of (A) TNF-α, (B) IL-6, and (C) MCP-1 expression in BALF. The data are presented as the mean ± standard deviation. ^*^P<0.05 and ^**^P<0.01 vs. the sham group. TNF-α, tumor necrosis factor α; IL-6, interleukin-6; MCP-1, monocyte chemoattractant protein-1; BALF, bronchoalveolar lavage fluid; ELISA, enzyme-linked immunosorbent assay; Dex, dexmedetomidine; Yoh, yohimbine; I/R, ischemia-reperfusion.

The ELISA analysis of BALF demonstrated that the concentration of TNF-α, IL-6 and MCP-1 in the I/R (784.27±57.78, 738.02±73.84 and 755.55±33.36, respectively) and Yoh (710.71±31.67, 784.43±59.18 and 781.65±59.98, respectively) groups were significantly higher compared with that of the sham group (315.58±54.38, 299.79±28.90 and 666.31±17.16, respectively) (all P<0.01) whereas the concentration of TNF-α, IL-6 and MCP-1 was not significantly different between the I/R and Yoh groups. The concentration of TNF-α and IL-6 in the Dex2.5 (509.21±55.10 and 531.56±49.97, respectively), Dex5 (488.13±35.93 and 484.94±39.96, respectively) and Dex+Yoh (574.92±70.29 and 500.68±33.98, respectively) groups were significantly lower compared with that of the I/R group (all P<0.01), whereas the concentration of MCP-1 in the Dex2.5 (711.42±26.68) and Dex+Yoh (697.89±22.21) groups was not significantly different compared with that of the I/R group (all P>0.05), although dexmedetomidine significantly inhibited the increase in MCP-1 concentration in the Dex5 group (685.95±37.88; P<0.05) compared with that in the I/R group (Fig. 3).

### Dexmedetomidine relieves LIRI-induced upregulation of TLR4 and MyD88 mRNA expression in the lung

**Figure 4:**
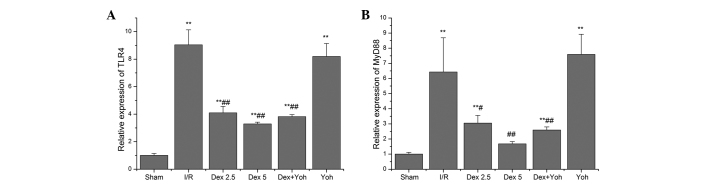
Expression of (A) TLR4 mRNA and (B) MyD88 mRNA in lung tissues harvested from rats of the six groups. The data are presented as the mean ± standard deviation. ^*^P<0.05 and ^**^P<0.01 vs. the sham group; ^#^P<0.05 and ^##^P<0.01 vs. the I/R group. TLR4, toll-like receptor-4; MyD88, myeloid differentiation factor 88; Dex, dexmedetomidine; Dex 2.5, 2.5 μg/kg/h Dex; Dex 5, 5 μg/kg/h; Yoh, yohimbine; I/R, ischemia-reperfusion.

The lung TLR4 mRNA expression of the I/R (9.04±1.09) and Yoh (8.20±0.94) groups was significantly upregulated following I/R compared with that of the sham group (1.01±0.14; P<0.01). Pre-treatment with dexmedetomidine at a dose of either 2.5 μg/kg/h (4.10±0.47; P<0.01, vs. the I/R group) or 5 μg/kg/h (3.29±0.13; P<0.01, vs. the I/R group) significantly inhibited the upregulation of TLR4 mRNA expression. However, yohimbine failed to reverse the downregulative effects of dexmedetomidine on TLR4 mRNA expression (Dex+Yoh group, 3.82±0.15; P<0.01, vs. the I/R group) (Fig. 4A).

The change in MyD88 mRNA expression in the lung was paralleled by that of the TLR4 mRNA expression (Fig. 4B). The MyD88 mRNA expression of the I/R (6.43±2.26) and Yoh (7.59±1.33) groups was significantly upregulated following I/R compared with that of the sham group (1.0±0.12; P<0.01). Pre-treatment with dexmedetomidine at a dose of either 2.5 μg/kg/h (3.05±0.51; P<0.05, vs. the I/R group) or 5μg/kg/h (1.68±0.15; P<0.01, vs. the I/R group) significantly inhibited the upregulation of MyD88 mRNA expression. However, yohimbine failed to reverse the downregulative effects of dexmedetomidine on MyD88 mRNA expression (Dex+Yoh group, 2.59±0.21; P<0.01, vs. the I/R group).

### Effects of dexmedetomidine on P-JNK and P-ERK

**Figure 5:**
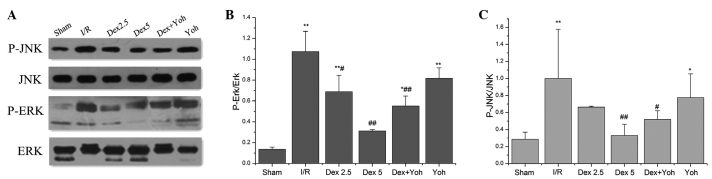
Activation of mitogen-activated protein kinase in lung tissues harvested from rats of the six groups. (A) Western blot analysis of JNK and ERK activation; (B) quantification of the p-JNK/JNK in (A); (C) quantification of the p-ERK/ERK in (A). The data are presented as the mean ± standard deviation. ^*^P<0.05 and ^**^P<0.01 vs. the sham group; ^#^P<0.05 and ^##^P<0.01 vs. the I/R group. I/R, ischemia-reperfusion; Dex, dexmedetomidine; Dex 2.5, 2.5 μg/kg/h Dex; Dex 5, 5 μg/kg/h; Yoh, yohimbine; P-JNK, phosphorylated c-Jun N-terminal kinase; P-ERK, phosphorylated extracellular signal-regulated kinase.

The levels of P-JNK in the I/R and Yoh groups were significantly higher following I/R compared with that in the sham group. Pre-treatment with dexmedetomidine at a dose of either 2.5 or 5 μg/kg/h significantly lowered the level of P-JNK compared with that in the I/R group. However, yohimbine failed to reverse the effect of dexmedetomidine on P-JNK, and yohimbine alone did not affect the level of P-JNK compared with that in the I/R group (Fig. 5).

The levels of P-ERK in the I/R and Yoh groups were significantly higher following I/R compared with that in the sham group. Pre-treatment with dexmedetomidine at a dose of 5 μg/kg/h significantly lowered the level of P-ERK compared with that in the I/R group, however, yohimbine failed to reverse its effect on P-ERK. Notably, pre-treatment with dexmedetomidine at a dose of 2.5 μg/kg/h and yohimbine alone did not affect the level of P-ERK compared with that in the I/R group (Fig. 5).

## Discussion

LIRI is a complex phenomenon involving intracellular injury processes, uncontrolled inflammatory processes and biochemical changes. Human trials and animal experiments have provided important descriptive information about the onset and evolution of the physiological and inflammatory changes in the lung. A body of evidence explains the possible mechanisms of LIRI and multiple interventions, including pharmacological treatments, such as adenosine α_2_ A agonist (17) and diazoxide (18), and other interventions, including ischemic pre-conditioning (19) and ischemic postconditioning (IPO) (20), have been administered to relieve lung damage induced by I/R injury, which could compromise the full benefit of reperfusion following ischemia. In the present study, pre-administration of dexmedetomidine at a dose of 2.5 or 5 μg/kg/h relieved the lung damage observed in the histological findings, reduced the lung w/d weight ratio and MPO activity and decreased the concentration level of inflammatory molecules, including TNF-α, IL-6 and MCP-1, in BALF. Furthermore, the TLR4/MyD88/MAPK signaling pathway was observed to be involved in the mechanism of the protective effect of dexmedetomidine on the lung following I/R injury, although an α_2_-adrenergic antagonist failed to neutralize the effect of dexmedetomidine on acute ischemia-induced lung injury.

High water content of the lung is a representative symptom of acute lung injury. The present results demonstrated that pre-administration of dexmedetomidine attenuated the development of pulmonary edema, as indicated by the significant decrease in the lung w/d weight ratio compared with the I/R group, although the ABG result did not reveal a significant difference between the pre-conditioned dexemdetomidine groups and the I/R group. In addition, pre-treatment with dexmedetomidine suppressed the lung MPO activity, which is a marker enzyme of neutrophils and is released from azurophilic granules of neutrophils. The decrease in MPO activity directly reflected the reduced infiltration of neutrophils in the lung, contributing to reducing lung damage. Dexmedetomidine also improved the lung histological examination. These findings highlighted the potential protective effect of dexmedetomidine on pulmonary injury induced by I/R, besides the protection against brain I/R injury reported in previous studies (21–24).

It is known that an increase in the level of pro-inflammatory cytokines, including TNF-α and IL-6, is an early feature of acute lung injury induced by clinical and experimental I/R. The present data demonstrated that dexmedetomidine decreased the concentrations of TNF-α, IL-6 and MCP-1 in BALF compared with that of the I/R group. This anti-inflammatory action of dexmedetomidine has been documented in previous studies. Nishina *et al* (25) first reported that clinically relevant concentrations of dexmedetomidine did not affect the chemotaxis, phagocytosis or superoxide production by human neutrophils or the intracellular calcium concentrations in neutrophils stimulated by chemotaxin *in vitro*, indicating that special precautions may not be required when using dexmedetomidine in patients with infection, sepsis or systemic inflammation. Subsequently, a body of animal and clinical trials (26–29) reported that dexmedetomodine reduced the level of plasma cytokines, including TNF-α, IL-1 and IL-6, stimulated by endotoxemia, and that dexmedetomidine reduced the mortality rate in endotoxemia-induced shock rat models in a dose-dependent manner. In a lipopolysaccharide-induced acute lung injury model (13), dexmedetomidine improved congestion and edema and reduced the w/d weight ratio and TNF-α, IL-1β and IL-6 levels in lung tissues. The present study further revealed that dexmedetomidine may be involved in the protection against sterile acute lung injury.

TLR4 is a transmembrane protein that is expressed in alveolar macrophages, endothelial cells, monocytes and neutrophils, and recognizes pathogen-associated molecular patterns and DAMPs (30–32). A growing body of evidence links TLR4/MyD88 signaling to the deleterious inflammatory effects observed in organs following I/R injury (33–37). In the LIRI mouse model, TLR4^−/−^ mice demonstrated a reduction in vascular permeability, lung MPO activity and the levels of several proinflammatory cytokines/chemokines in BALF samples compared with those from wild-type mice. In accordance with that result, upregulation of TLR4 mRNA was observed in the lung in the I/R group compared with that in the sham group, and pre-treatment with dexmedetomidine downregulated the expression of TLR4 and MyD88 mRNA, as the downstream signal molecule of TLR4 in the lung, compared with that in the I/R group. Correspondingly, the change trends of the TNF-α, IL-6 and MCP-1 concentrations in BALF were consistent with the TLR4 and MyD88 mRNA expression in the lung, indicating that TLR4/MyD88 signaling may be involved in the anti-inflammatory mechanism of dexmedetomidine to inhibit LIRI.

TLR4/MyD88 signaling ultimately leads to the activation of NF-κB and certain MAPKs, e.g., p38, ERK1/2 and JNK, which result in the production of inflammatory cytokines, TNF-α and IL-6, following I/R injury (38,39). The present study demonstrated that LIRI increased the phosphorylation of JNK and ERK rather than p38 (data not shown) in the I/R group compared with the sham group. Pre-treatment with dexmedetomidine reduced the expression of P-JNK and P-ERK1/2 proteins in the lung compared with those in the I/R group. However, in an intestinal I/R-induced remote lung injury model, inhibition of p38 activation has been shown to alleviate neutrophil infiltration and lung cytokine expression rather than JNK or ERK1/2 (38). Zanotti *et al* (39) reported that functioning TLR4 results in the early phosphorylation of p38 observed during ischemia and the early phosphorylation of ERK and JNK following reperfusion, associated with inflammation rather than lung w/d weight ratio in mouse LIRI. The difference in the MAPK activity between the data of the present study and previous results may be attributable to the difference in the models, the animals or the observation time.

Notably, administration of the α_2_-adrenoceptor antagonist, yohimbine, prior to dexmedetomidine pre-treatment failed to completely eliminate the effect of dexmedetomidine on TLR4 expression, the phosphorylation of JNK and ERK and the production of inflammatory cytokines in BALF, indicating that the anti-inflammatory mechanism of dexmedetomidine may be associated with an α_2_-adrenoceptor-independent signaling pathway, although dexmedetomidine may attenuate the excessive release of plasma noradrenaline induced by ischemia by the activation of presynaptic α_2_-adrenoceptor, contributing to the relief of the inflammatory response. Gu *et al* (7) demonstrated that dexmedetomidine markedly reduces renal I/R induced pulmonary injuries and lowers the MPO activity and cytokine expression, but the α_2_-adrenoceptor antagonist, atipamezole, partially reverses the protective effects of dexmedetomidine in the lung and has no effect on the cytokine expression level. In addition, by acting through the I1 imidazoline receptor, dexmedetomidine has been shown to exhibit a pattern of neuroprotective action in hippocampal slices obtained from rats (40), while its action through the I2 imidazoline receptor has been shown to reduce cell apoptosis and necrosis to protect against oxygen-glucose deprivation-induced injury in rat C6 cells in an *in vitro* model of ischemia (41). The activation of the varying receptors induced by dexmedetomidine may play a protective role through a different mechanism to that of organ I/R injury.

Several limitations exist for the present study. This study lacked analysis of the time-associated effects of dexmedetomidine on the inflammatory response to LIRI. Therefore, the possibility that dexmedetomidine affected the extent of expression of various inflammatory cytokines and proteins may not be ruled out. In addition, dexmedetomidine has an analgesic effect (42). By contrast, no analgesic was applied in the I/R group. Therefore, the question of whether the analgesic peculiarity of dexmedetomidine is likely to play a role remains to be elucidated. Furthermore, the effect of dexmedetomidine on the activation of NF-κB following LIRI was not examined, therefore the possibility that TLR4/NF-κB signaling is involved in the anti-inflammatory mechanism of dexmedetomidine against LIRI may not be ruled out.

The present study demonstrated that lung I/R upregulated TLR4 and MyD88 mRNA expression and P-JNK and P-ERK1/2 protein expression in lung tissue, and increased the concentration levels of TNF-α, IL-6 and MCP-1 in BALF. Pre-administration of dexmedetomidine prior to I/R reduced pulmonary damage in the histological results and decreased MPO activation in lung tissue and the concentration levels of proinflammatory cytokines in BALF through the TLR4/MyD88/MAPK signaling pathway. Our study provides a potential clinical application of dexmedetomidine for reducing lung ischemia-reperfusion injury in an experimental model.

## Figures and Tables

**Table I:** ABG data.

Group (n=12)	pH	PaO_2_, mmHg	PaCO_2_, mmHg	Base excess, mM
Sham	7.19±0.22	139.3±28.4	26.0±5.4	−16.5±8.0
I/R	7.23±0.16	119.5±22.2	31.0±6.2	−13.1±5.8
Dex2.5	7.27±0.09	123.5±20.4	27.5±6.9	−13.1±3.7
Dex5	7.21±0.13	138.8±31.0	27.2±4.7	−15.3±5.1
Dex+Yoh	7.28±0.07	128.7±11.7	27.7±4.2	−11.8±1.9
Yoh	7.27±0.06	116.8±27.4	29.3±4.1	−12.8±2.1

Data are presented as the mean ± standard deviation. ABG, arterial blood gas; I/R, ischemia-reperfusion; Dex, dexmedetomidine; Dex 2.5, 2.5 μg/kg/h Dex; Dex 5, 5 μg/kg/h; Yoh, yohimbine.
